# Fungal cell barriers and organelles are disrupted by polyhexamethylene biguanide (PHMB)

**DOI:** 10.1038/s41598-023-29756-w

**Published:** 2023-02-16

**Authors:** Winnie Ntow-Boahene, Isabelle Papandronicou, Josephous Miculob, Liam Good

**Affiliations:** Pathobiology and Population Sciences, The Royal Veterinary College, London, England

**Keywords:** Biological techniques, Cell biology, Microbiology

## Abstract

The similarities between fungal and mammalian cells pose inherent challenges for the development of treatments for fungal infections, due to drug crossover recognition of host drug targets by antifungal agents. Thus, there are a limited number of drug classes available for treatment. Treatment is further limited by the acquisition and dissemination of antifungal resistance which contributes to the urgent need of new therapies. Polyhexamethylene biguanide (PHMB) is a cationic antimicrobial polymer with bactericidal, parasiticidal and fungicidal activities. The antifungal mechanism of action appears to involve preferential mechanical disruption of microbial cell structures, offering an alternative to conventional antifungals. However, the antifungal mechanisms have been little studied. The aim of this study was to characterise PHMB’s activities on selected yeast (*Saccharomyces cerevisiae, Candida albicans*) and filamentous fungal species (*Fusarium oxysporum, Penicillium glabrum*). Fungal membrane disruption, cell entry and intracellular localisation activities of PHMB were evaluated using viability probe entry and polymer localisation studies. We observed that PHMB initially permeabilises fungal cell membranes and then accumulates within the cytosol. Once in the cytosol, it disrupts the nuclear membrane, leading to DNA binding and fragmentation. The electrostatic interaction of PHMB with membranes suggests other intracellular organelles could be potential targets of its action. Overall, the results indicate multiple antifungal mechanisms, which may help to explain its broad-spectrum efficacy. A better understanding of PHMB’s mechanism(s) of action may aid the development of improved antifungal treatment strategies.

## Introduction

Despite the increasing rates of invasive fungal infections, there are only five distinct chemical classes of antifungals used clinically: azoles, echinocandins, polyenes, pyrimidine analogues and allylamines^1^. These drugs are associated with numerous limitations making them inadequate for tackling certain emerging fungal infections. Subsequently, they do not fulfil clinical needs as treatment outcomes remain unfavourable^2^. Associated limitations include poor bioavailability, the biochemical overlap between fungal pathogens and host, as well as the emergence of resistance^3,4^. The emergence of increasing resistance is driven in part, by the antifungal mechanism of these classes as each class primarily inhibits a single cellular target with fungistatic or fungicidal outcomes^2^. In addition, some fungal species show inherent reduced susceptibility to some antifungal drugs, such as *C. glabrata* and *C. krusei* resistance to fluconazole^4^. Another limitation is accessibility due to the route of administration of these drugs. For example, the echinocandin class achieves poor oral bioavailability due to their chemical properties such as high molecular weight. To circumvent this, they are administered intravenously daily which is not viable as a long-term treatment option in many situations^5^. Thus, these existing classes of antifungal agent do not meet the unmet clinical need of fungal infections, especially when more serious invasive infections are considered.

Various cationic antimicrobial polymers are currently in development or already used clinically due to their ability to kill a broad-spectrum of microorganisms through electrostatic interactions of their active groups with the microbial surface^6^. Examples of active cationic groups include ammonium groups, halamines, biguanides or poly lysine^7^. Acquired antimicrobial resistance to these agents has not been observed in fungi despite prolonged use of these polymers. This could be attributed to the non-specific mechanism against cell barriers. Therefore, the use of antimicrobial polymers could provide a potentially superior strategy in the race to find potent antifungal solutions. However, such general cell disruptive properties also raise toxicological concerns about general cytotoxic effects on host cells. Different cationic groups appear to yield distinct effects on microbial and host cells. For example, guanidine polymers were shown to be more potent against *S. epidermidis*, methicilin-resistant *Staphylococcus aureus* (MRSA), *E. coli* and *C. albicans* whilst being less toxic to human keratinocyte cells in comparison to amine polymers^7^.

Polyhexamethylene Biguanide (PHMB) is a synthetic cationic polymer consisting of repeating biguanide units and has been established as an effective antimicrobial agent against bacteria and fungi^9,10^. It demonstrates a high therapeutic index with broad-spectrum antimicrobial activity due to its biguanide groups with no reports of acquired antimicrobial resistance^7,11^. The currently accepted model for the antibacterial activity of PHMB against bacterial species is via microbial membrane permeabilisation, where PHMB selectively kills microbial cells by forming pores in microbial cell membranes through interaction with phospholipids^12–14^. In comparison to microbial cells, PHMB demonstrated relatively less activity with the cell membrane glycoproteins on mammalian cell membranes, explaining the polymer’s high therapeutic index^14,15^. However, the membrane disruption model fails to explain the induction of DNA repair pathways in *E. coli*, following exposure to PHMB^16^. A proposed alternative mechanism of action could involve energy mediated cell entry of PHMB into microbial cells, to inhibit intracellular targets. This mechanism was observed for *Bacillus megaterium*, where PHMB localised within the cytoplasm, with no detectable cell membrane damage^17^.

In the case of fungi, knowledge of the mechanism of action of PHMB is limited, but a similar mechanism involving cell wall destabilisation has been proposed^18,19^. The *β*-glucan structure of the *S. cerevisiae* cell wall was reported to be the target for PHMB disruption where gene expression studies indicated an increase in the expression of cell wall integrity genes (CWI) and protein kinase C (PKC) for cell maintenance during arduous environmental conditions^18,19^.

In this present study we explore the antifungal mode of action of PHMB against selected fungal species *Saccharomyces cerevisiae* (S288c; ATCC), *Fusarium oxysporum, Penicillium glabrum* and *Candida albicans* R1 as they represent a selection of common pathogenic yeast and filamentous fungi. We demonstrate that PHMB initially permeabilises fungal cell membranes and then accumulates within the cytosol as previously seen with bacteria and mammalian cells^17^. PHMB does not remain trapped within endosomes as seen with mammalian cells. Instead, it escapes and disrupts the fungal nuclear membrane; resulting in condensation of fungal chromosomes and cell death. In addition to the nuclear membrane, PHMB may disrupt the membranes of other organelles. In contrast, PHMB remains trapped within endosomes in mammalian cells, suggesting that the polymer distinguishes between microbial and non-microbial eukaryotic cell structures. Overall, the results help to explain the antifungal mechanisms of PHMB and its selective toxicities against eukaryotic microbes.

## Materials and methods

### Fungal strains and growth conditions

The fungal strains were *Saccharomyces cerevisiae* (S288c; ATCC), *Fusarium oxysporum, Penicillium glabrum* and *Candida albicans* R1 (S. Kelly; University of Sheffield). Fungi were grown first on plates using Sabouraud Dextrose Agar (SDA) at 30 °C for 48 h. For overnight cultures, single yeast colonies were transferred to liquid culture (RPMI-1640 media (Sigma) supplemented with 2% glucose). Filamentous fungi were rinsed in 3 ml of RPMI-1640 media supplemented with 2% glucose for spore collection and grown at 30 °C overnight.

### Minimum inhibitory concentration (MICs)

PHMB and PHMB labelled with rhodamine (PHMB–rhodamine) were obtained from Tecrea Ltd, UK and stock solutions were made in sterile dH_2_O. Terbinafine (Sigma-Aldrich) stock solutions were made in 80% ethanol. To determine a suitable concentration range for the membrane permeabilisation assays, the minimum inhibitory concentrations of PHMB, negative control (Terbinafine) and positive control (Triton x-114) were determined against all fungal species in RPMI-1640, 2% glucose using the broth microdilution method^20,21^. A 96 well microplate with serial dilutions of drug was inoculated with fungi (*S. cerevisiae*, *F. oxysporum, P. glabrum* and *C. albicans* R1) at 1 × 10^4^ cells/ml. Plates were incubated at 30 °C for 48 h and absorbance was measured at OD600nm. The lowest concentration of PHMB that inhibited ~ 90% fungal growth was determined as the MIC_90_ and ~ 50% growth was determined as the MIC_50_ respectively.

## Time dependent PHMB permeabilisation of fungal cell membranes at MIC_50_ and sub-MIC concentrations

100 µl of PHMB, terbinafine and Triton x-114 were added to wells of a 96 microwell plate containing fungi (*S. cerevisiae*, *F. oxysporum, P. glabrum* and *C. albicans* R1) at 1 × 10^4^ cells/ml at their final MIC_50_ concentration. Fungi were heat killed as a positive control of maximum relative fluorescence units (RFU) following complete lysis. Fungal cultures were transferred to a glass test tube and then heat-killed with a Bunsen burner flame for 10 s. Triton x-114 (Sigma Aldrich) was used as a positive control of maximum RFU for complete membrane permeabilisation. Terbinafine, an antifungal with no membrane permeabilisation activity was used as a negative control for cell death with no membrane permeabilisation. SYTOX Green (Molecular probes), a membrane permeable DNA binding agent was added to each well to a final concentration of 8 µM. Fluorescence intensity was measured using a Tecan M200 Infinite Pro Microplate Reader with Magellan software version 7.0, at 485 nm excitation/ 520 nm emission every 15 min for 3 h at 30 °C.

### Concentration dependent PHMB permeabilisation of fungal cell membranes

Serial dilutions of PHMB were performed with the highest concentration of 32.4 µg/ml were added to fungal cells (*S. cerevisiae*, *F. oxysporum, P. glabrum* and *C. albicans* R1) at 1 × 10^4^ cells/ml in a 96 well microplate. 8 µM SYTOX Green (Thermofisher) was also added to the plate and incubated for 3 h. Fluorescence measurements were taken after 3 h.

### SYTOX Green fluorescence imaging of PHMB membrane permeabilisation in *S. cerevisiae* at sub-MIC

Serial dilutions of PHMB were performed with the highest concentration of 4.05 µg/ml were added to *S. cerevisiae* cultures (1 × 10^4^ cells/ml) and incubated with 8 µM SYTOX Green (Thermofisher) for 3 h at 30 °C. Samples were centrifuged at 12,000 rpm for 5 min. The supernatant was discarded, and the cell pellet was washed with PBS. Cells were imaged using a Leica DMIRB inverted microscope with Axiovision Rel. 4.8 software (Zeiss) and 40 × objective lens using the greenband pass filter and phase contrast.

### Fluorescent labelling of PHMB

PHMB labelled with rhodamine were obtained from Tecrea Ltd, UK (PHMB-rhodamine). To determine no loss of antifungal function of PHMB following labelling, a lawn culture of *S. cerevisiae* was prepared on SD agar and incubated with 10 µl spots of 1 mg/ml PHMB (Tecrea Ltd.) and 1 mg/ml PHMB-rhodamine 30 °C for 48 h.

### Confocal microscopy of PHMB-rhodamine localisation within fungi

*S. cerevisiae* cultures were treated with PHMB-rhodamine at 4 μg/ml and 8 μg/ml. *C. albicans* aliquots were treated at 8 μg/ml and 12 μg/ml. Samples were protected from light during incubation steps. Untreated controls were treated with RPMI 1640, 2% glucose. Samples were incubated at room temperature for 4 h and centrifuged at 12,000 rpm for 5 min. The resulting supernatant was discarded, and the cell pellet fixed with 50 μl of 4% paraformaldehyde (PFA) in PBS for 15 min. Samples were resuspended with PBS and centrifuged at 12,000 rpm for 5 min. The supernatant was discarded, and the cell pellet was resuspended in PBS prior to counter-staining with 50 μg/ml of Concanavalin A conjugated with Alexa Fluor 488 (Thermofisher) and 10 μl Prolong Diamond Antifade Mountant with DAPI (Thermofisher). All samples were mounted on microscope slides with coverslips and sealed with nail varnish. Slides were stored at 4 °C protected from light. Confocal images were taken with a Leica SP5 confocal microscope. Sequential Z-stacks for *S. cerevisiae* and *C. albicans* (slice numbers of 59 and 35, respectively) were collected using a line average of 64 (256 × 256, zoom factor 8). and Super Resolution Radial Fluctuations (SRRF) analysis was performed by taking 1000 frames (256 × 256, zoom factor 8) at a line average of 1 and processed using ImageJ version 1.52i.

### Analysis of PHMB exposure on fungal membrane integrity through Con A-Alexa Fluor 488 fluorescence quantification

*S. cerevisiae* was treated with PHMB-rhodamine at 4 µg/ml and 8 µg/ml and *C. albicans* was treated with 8 µg/ml and 12 µg/ml. All samples were incubated at 30 °C for 4 h, protected from light. Untreated controls were treated with the same volumes of RPMI-1640, 2% glucose. Samples were fixed and counterstained as previously described for fluorescence microscopy. The Con A-Alexa Fluor 488 membrane fluorescence intensity of sampled cells (n = 20) were measured at four symmetrical points along the cell membrane. Data points = (mean ± SD).

### Statistics

Fluorescence intensity data were analysed by Repeated Measures (RM) One-way or Two-way Analysis of Variance (ANOVA) followed by Tukey’s multiple comparison test using GraphPad Prism 7 software. Significance = *p* < 0.05.

## Results

### Minimum inhibitory concentrations (MICs) of PHMB against fungi

To determine a suitable concentration range for the membrane permeabilisation assays, the minimum inhibitory concentrations of PHMB, negative control (Terbinafine) and positive control (Triton x-114) were determined against *S. cerevisiae*, *C. albicans, F. oxysporum* and *P. glabrum* as representative species of yeast and filamentous fungi. PHMB-rhodamine MIC_90_ was also determined and compared to unlabelled PHMB to confirm no loss of antifungal activity following labelling. Yeast cells appear to be more susceptible to PHMB attack than filamentous fungi as a lower concentration of PHMB is required for growth inhibition (Table 1). In this study, terbinafine MIC_s_ for *C. albicans* R1 and *S. cerevisiae* is slightly lower than reported values^22,23^. However, MICs observed for *F. oxysporum* and *P. glabrum* agrees with ranges reported for the fungal species tested^24,25^.

**Table 1:** Minimum inhibitory concentrations (MICs) of PHMB, PHMB-rhodamine, Terbinafine and Triton x-114 against selected yeast and filamentous fungi.

Species	PHMB MIC_50_ (µg/ml)	PHMB MIC_90_ (µg/ml)	PHMB-rhodamine MIC_90_ (µg/ml)	Terbinafine MIC_50_ (µg/ml)	Triton x-114 MIC_50_ (µg/ml)
*S. cerevisiae*	1	2	4	0.84	1.17
*C. albicans*	1–2	4	8	0.84	1.17
*F. oxysporum*	2	4–8	–	3.4	4.7
*P. glabrum*	2	4–8	–	1.7	2.5

### PHMB permeabilisation of fungal cell membranes at MIC and sub-MIC concentrations is time dependent

**Figure 1:**
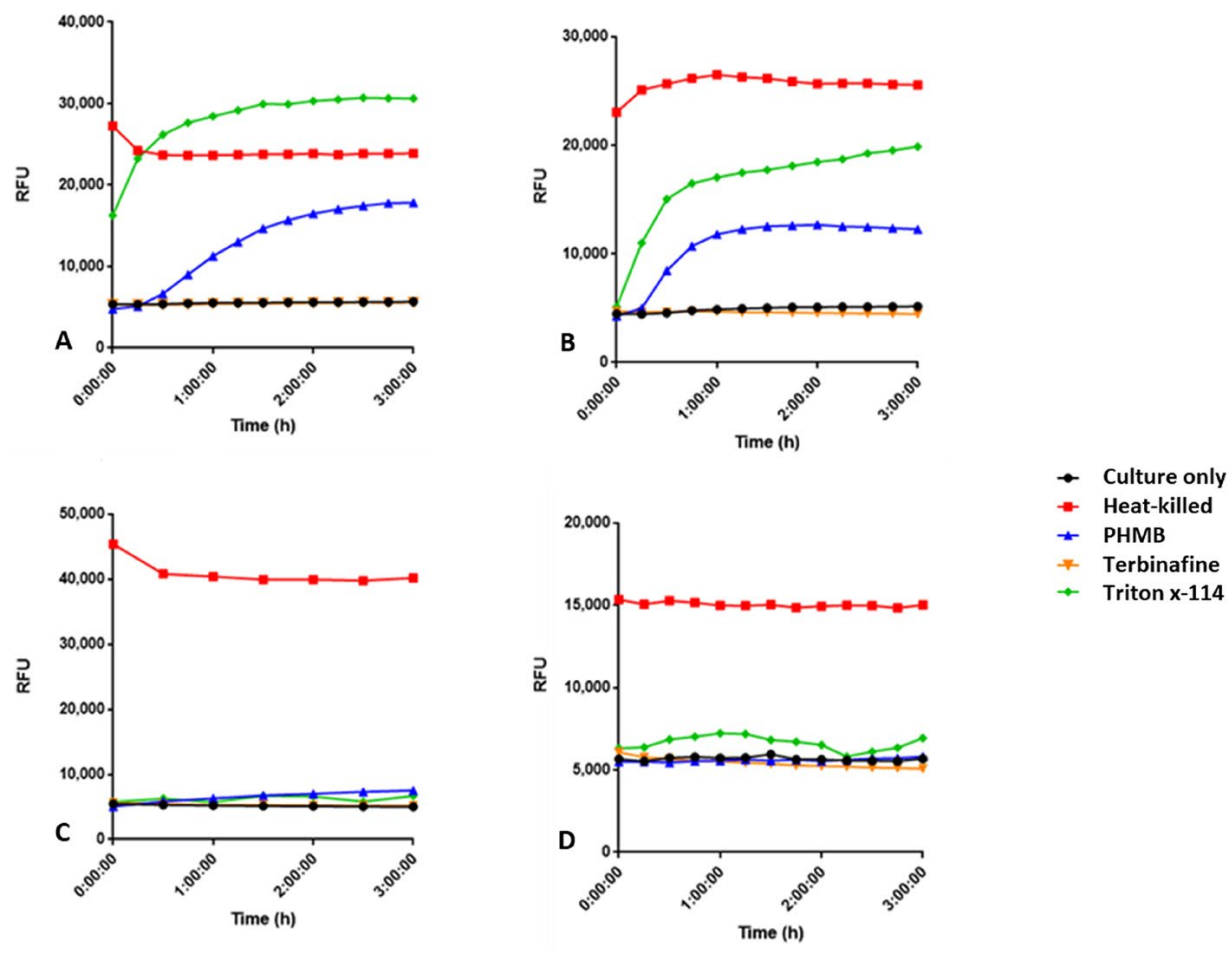
The effect of time on PHMB associated membrane permeabilisation. Fungi were treated with PHMB [MIC_50_] and SYTOX Green (8 µM). Fluorescence profiles for each species treated with PHMB are shown, with positive (heat killed; triton x-114) and negative (Terbinafine; untreated) controls. (**A**) *S. cerevisiae* heat-killed or treated with 1 µg/ml PHMB, 0.84 µg/ml Terbinafine, 1.17 µg/ml Triton x-114 (**B**) *C. albicans* R1 heat-killed or treated with 1 µg/ml PHMB, 0.84 µg/ml Terbinafine, 1.17 µg/ml Triton x-114 (**C**) *F. oxysporum* heat-killed or treated with 2 µg/ml PHMB, 3.84 µg/ml Terbinafine, 4.7 µg/ml Triton x-114 (**D**) *P. glabrum* heat-killed or treated with 2 µg/ml PHMB, 1.7 µg/ml Terbinafine, 2.5 µg/ml Triton x-114.

To assess the level of PHMB permeabilisation of fungal cell membranes, a viability stain assay was used with four species (*S. cerevisiae*, *F. oxysporum, P. glabrum* and *C. albicans* R1). The viability probe SYTOX Green is excluded from healthy cells with intact membranes but can enter cells after cell membrane damage. Upon cell entry, SYTOX Green binds DNA producing an increase in green fluorescence yield, above the baseline fluorescence. The antifungal terbinafine was used as a negative control to demonstrate antifungal efficacy in the absence of membrane disruption. As positive controls, cells were heat-killed or Triton X-114 treated. The culture only negative control displayed the baseline fluorescence of SYTOX Green. For all fungal species, the culture only control remained constant over time at approximately 4000—5000 relative fluorescence units (RFU) (Fig. 1). Only a slight increase in fluorescence, above the baseline fluorescence, was observed for the terbinafine negative control in each fungal species. The heat-killed positive control displayed substantial increases in fluorescence at time 0 for each species which remained relatively constant over time. The Triton X-114 positive fluorescence control, also showed substantial increases in fluorescence for *S. cerevisiae* and *C. albicans* at time 0. However, for *F. oxysporum* and *P. glabrum.*, the Triton X-114 positive controls only showed a slight increase in fluorescence.

Similarly, PHMB MIC_50_ treated *S. cerevisiae* and *C. albicans* showed substantial increases in fluorescence at 15 min, indicating membrane permeabilisation. However, for the filamentous fungi, *F. oxysporum* showed a slight increase in fluorescence and *P. glabrum* showed no increase in fluorescence following PHMB treatment, suggesting minimal to no cell permeabilisation.

### PHMB permeabilisation of fungal cell membranes is concentration dependent

**Figure 2:**
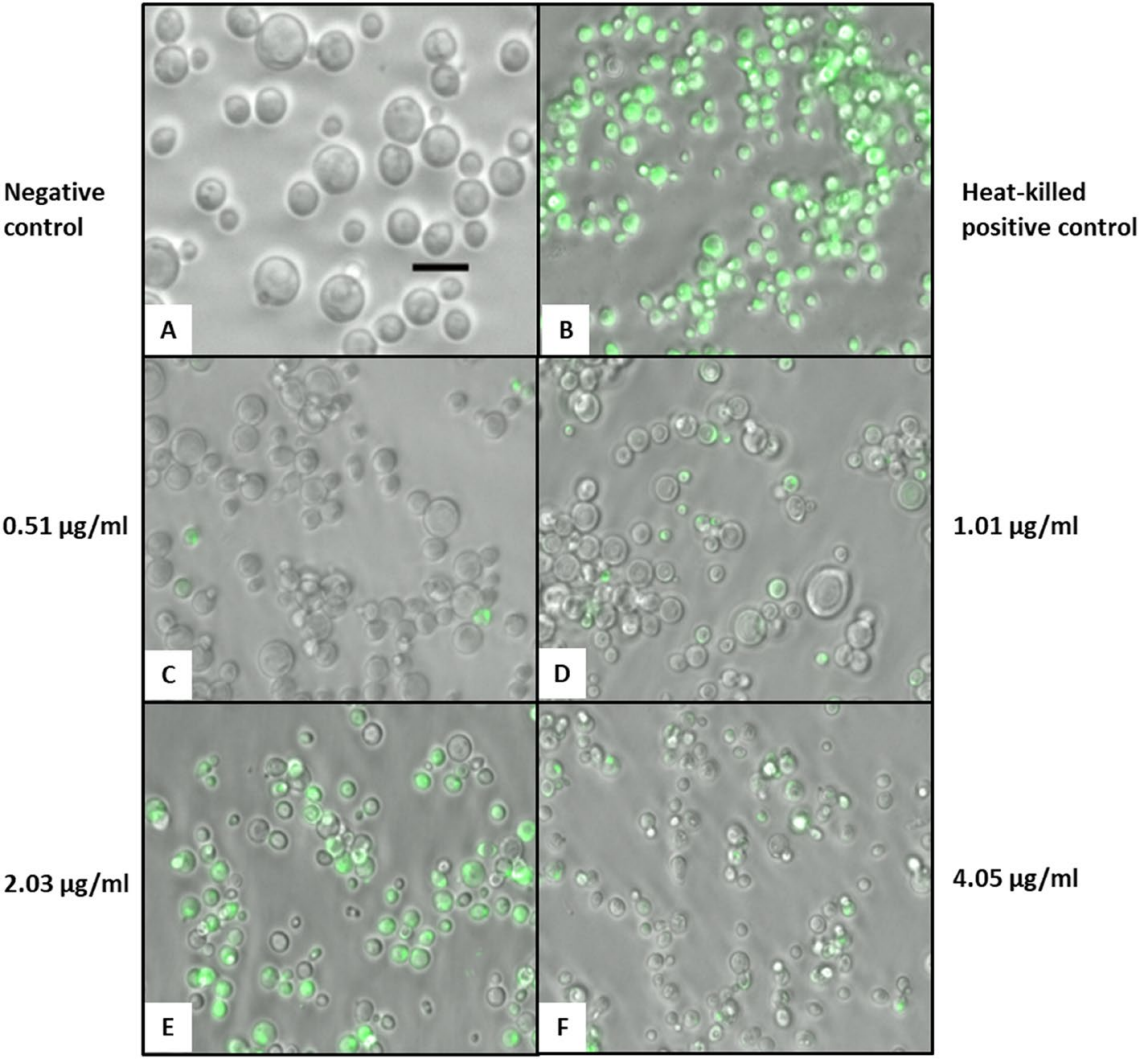
Fluorescence imaging showing the effect of PHMB on membrane permeability to SYTOX Green. SYTOX Green (8 µM) and varying PHMB concentrations were added to growth medium before 3 h incubation with *S. cerevisiae*. Live cell images were merged following imaging by phase contrast and green bandpass filter. Scale bar = 10 µm.

To visualise PHMB membrane permeabilisation and subsequent SYTOX green fluorescence, live *S. cerevisiae* cells were treated with PHMB for 3 h, and then examined using fluorescence microscopy imaging. Untreated cells showed no detectable fluorescence. Control heat-killed positive samples showed strong cell-associated SYTOX green fluorescence in most cells. Cell membrane permeabilisation/SYTOX green fluorescence increased with ascending concentrations of PHMB (from 0.51 to 2.03 µg/ml) which closely corresponds to MIC values (Fig. 2). At PHMB concentrations above the MIC_90_, the proportion of cells showing fluorescence and the strength of fluorescence decreased, suggesting that PHMB interacts with DNA inside cells, blocking subsequent binding by SYTOX green.

**Figure 3:**
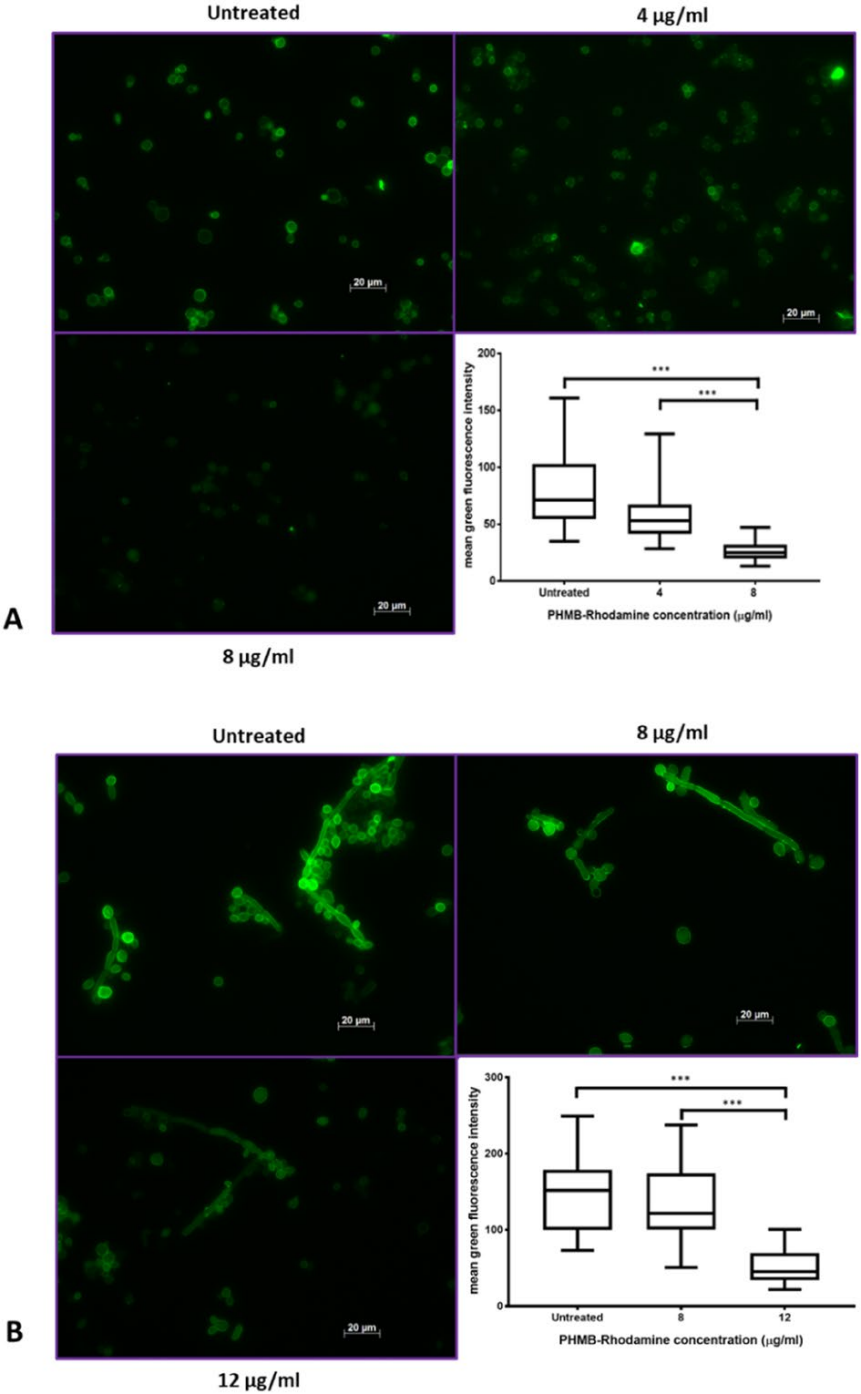
Fluorescence imaging showing the reduction of Con A- membrane fluorescence following exposure to increasing concentrations of PHMB. (**A**) *S. cerevisiae* cultures were treated with PHMB-rhodamine at 4 µg/ml and 8 µg/ml (**B**) *C. albicans* cultures were treated with 8 µg/ml and 12 µg/ml. Cultures were incubated at room temperature for 4 h and counter-stained with Con A-Alexa Fluor 488. Untreated control = growth media only. Images show quenching of membrane fluorescence intensity with increasing PHMB concentration. Graphs show measured fluorescence intensity of sampled cells (n = 20) at four symmetrical points along the cell membrane and averaged (mean ± SD). Membrane fluorescence was analysed by RM One-way ANOVA followed by Tukey's multiple comparison test.

To determine the impact of PHMB exposure on fungal cell membrane integrity, *S. cerevisiae and C. albicans* were treated with PHMB-rhodamine at their respective MIC_90_ concentrations and above, then counterstained with Con A-Alexa Fluor 488. *S. cerevisiae* (MIC_90_ = 4 µg/ml, above MIC_90_ = 8 µg/ml) and *C. albicans* (MIC_90_ = 8 µg/ml, above MIC_90_ = 12 µg/ml). Membrane fluorescence was quantified by measuring four symmetrical points along the cell membranes of randomly selected cells (n = 20) using ImageJ (Fig. 3). Fluorescence intensity of membrane stained Con A-Alexa Fluor 488 decreased with increasing PHMB concentrations in both species.

**Figure 4:**
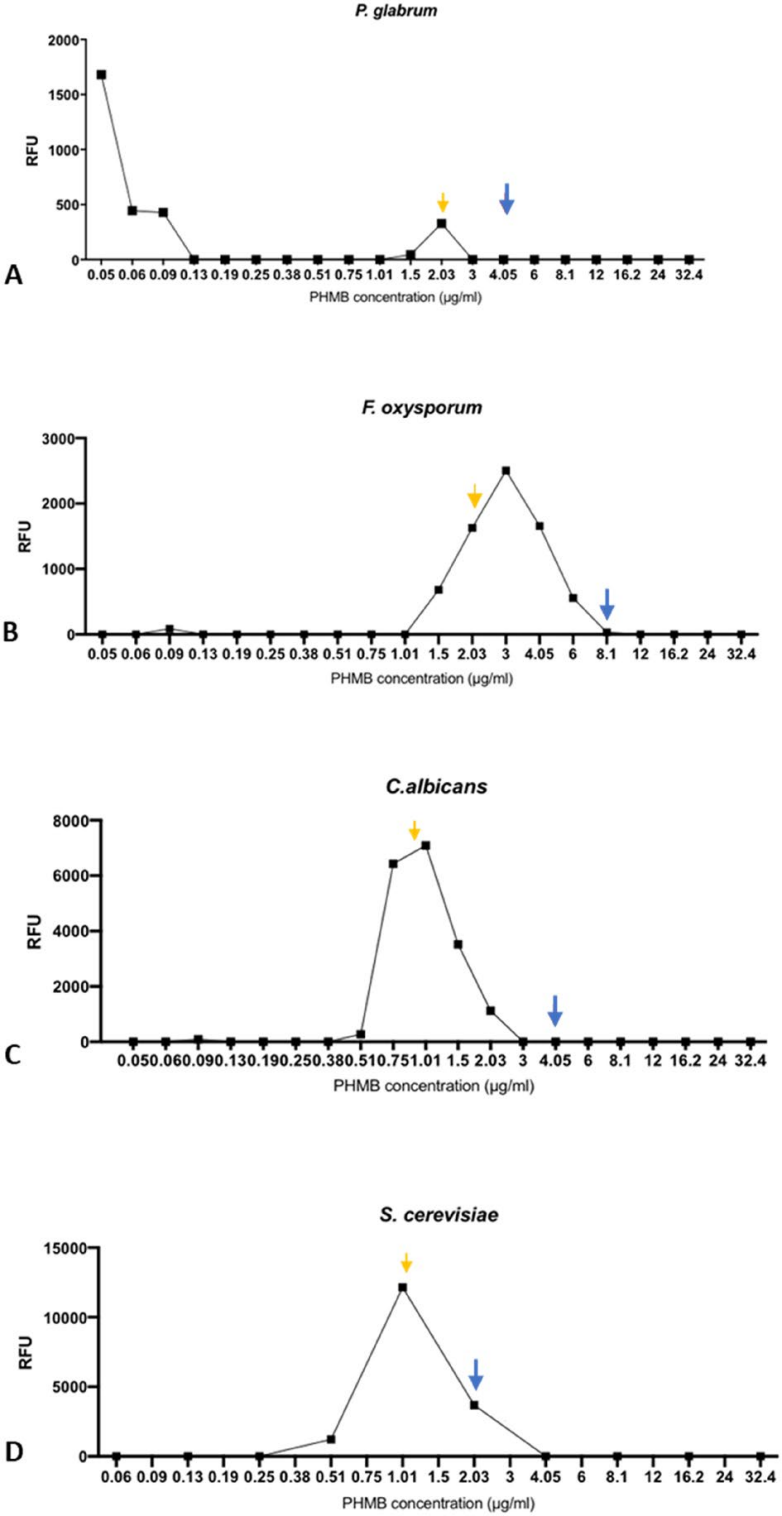
The effect of PHMB concentration on fungal cell membrane permeabilisation. SYTOX Green (8 µM) and PHMB concentrations were added to fungi (1 × 10^4^ cells/ml) in RPMI-1640, 2% glucose. Samples were incubated for 3 h, with fluorescence measurements taken after the incubation period. (**A**) *P. glabrum* (**B**) *F. oxysporum* (**C**) *C. albicans* (**D**) *S. cerevisiae*. Yellow arrow = MIC_50_ concentration. Blue arrow = MIC_90_ concentration.

To determine the effects of PHMB concentration on the extent of cell membrane permeabilisation, a range of PHMB concentrations up to 32 µg/ml were plotted against their fluorescence values after 3 h (Fig. 4). Fluorescence remained at background at subMIC_50_ for all fungal species. However, there was a concentration dependent increase in fluorescence which began at 0.25 µg/ml for *S. cerevisiae*, 0.38 µg/ml for *C. albicans* and 1.01 µg/ml for *F. oxysporum* with maximum fluorescence peaks occurring at or near PHMB MIC_50_ concentrations for *S. cerevisiae* (12,143 RFU, 1.01 µg/ml), *C. albicans* (7091 RFU, 1.01 µg/ml) and *F. oxysporum* (2,503 RFU, 3 µg/ml) due to increased cell membrane permeability. At PHMB concentrations above MIC_90_, there is a loss of SYTOX green fluorescence. As PHMB has the ability to bind DNA^16^, it likely outcompetes SYTOX green binding to quench the fluorescence signal. As PHMB permeabilisation appeared dependent on both concentration and time, the rate of PHMB uptake was calculated from the collected data. *P. glabrum* was excluded as no increase in cell membrane permeability/ SYTOX green fluorescence was previously observed.

### Rate of PHMB permeabilisation of fungal cell membranes

**Figure 5:**
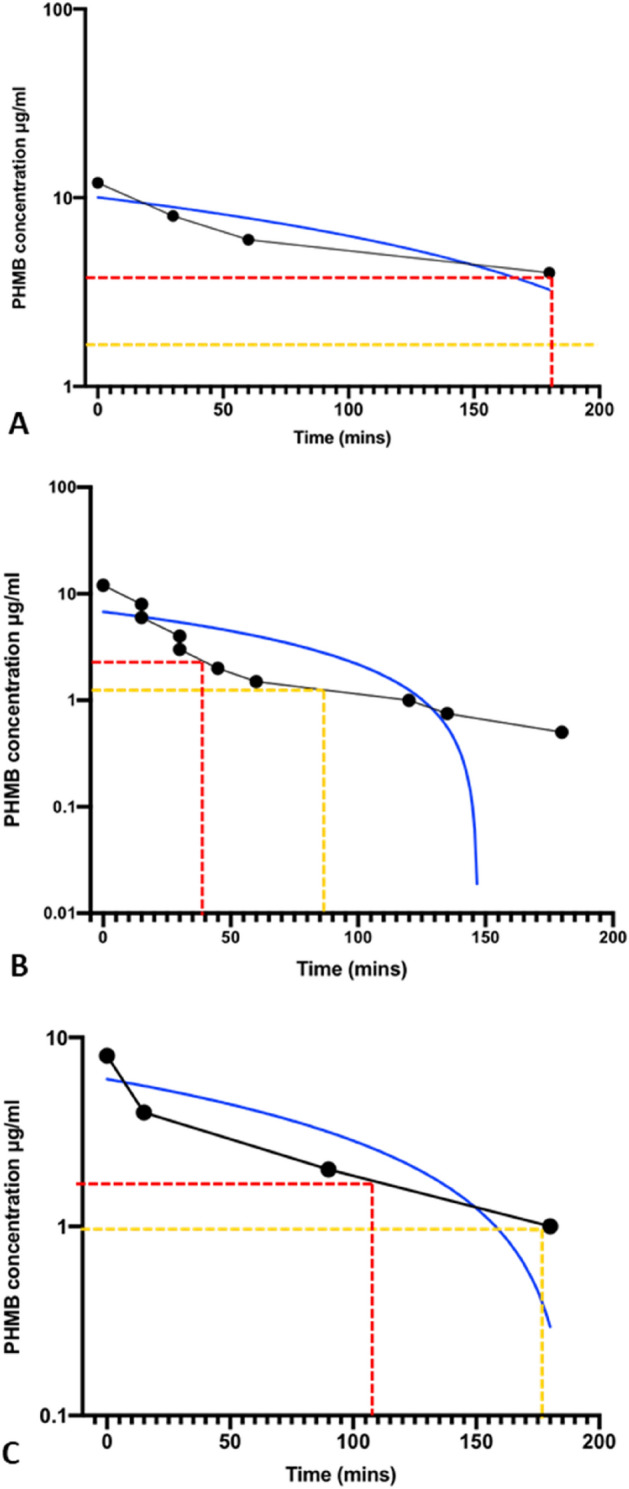
PHMB uptake rate by *F. oxysporum*, *S. cerevisiae* and *C. albicans*. The uptake was calculated to be 0.04 µg/ml min^−1^, 0.03 µg/ml min^−1^, 0.05 µg/ml min^−1^ respectively at concentrations of 8 µg/ml and above. (**A**) *F. oxysporum*, (**B**) *C. albicans*, **(C)** *S. cerevisiae*. Red line = MIC_90_ concentration , Yellow line = MIC_50_ concentration, Blue line = linear regression.

To determine rate of PHMB uptake into fungi, PHMB concentrations were plotted against their respective time taken for maximum cell membrane permeabilisation; where maximum cell membrane permeabilisation is the maximum fluorescence (RFU) produced due to SYTOX Green: DNA binding (Fig. 5). The uptake rate at 30^0^C was calculated to be 0.03775 μg/ml min^−1^, 0.03177 μg/ml min^−1^ and 0.04607 μg/ml min^-1^ for *F. oxysporum*, *S. cerevisiae* and *C. albicans* respectively (Table 2). At PHMB concentrations of 8 μg/ml and above, fungal cell membrane permeabilisation appears instantly with maximum RFU values achieved at time 0 for all species. For *F. oxysporum*, maximum permeabilisation is reached at ~ 180 min at MIC_90_ and 180 < min at MIC_50_ concentrations.

**Table 2:** PHMB uptake rate and time taken to maximum permeabilisation in various fungal species.

Fungi	Uptake rate (μg/ml min^−1^)	Time to maximum permeabilisation MIC_50_ (min)	Time to maximum permeabilisation MIC_90_ (min)
*S. cerevisiae*	0.03177	177	110
*C. albicans*	0.04607	85	40
*F. oxysporum*	0.03775	180 <	180

### PHMB localises internally within *S. cerevisiae* and *C. albicans*

**Figure 6:**
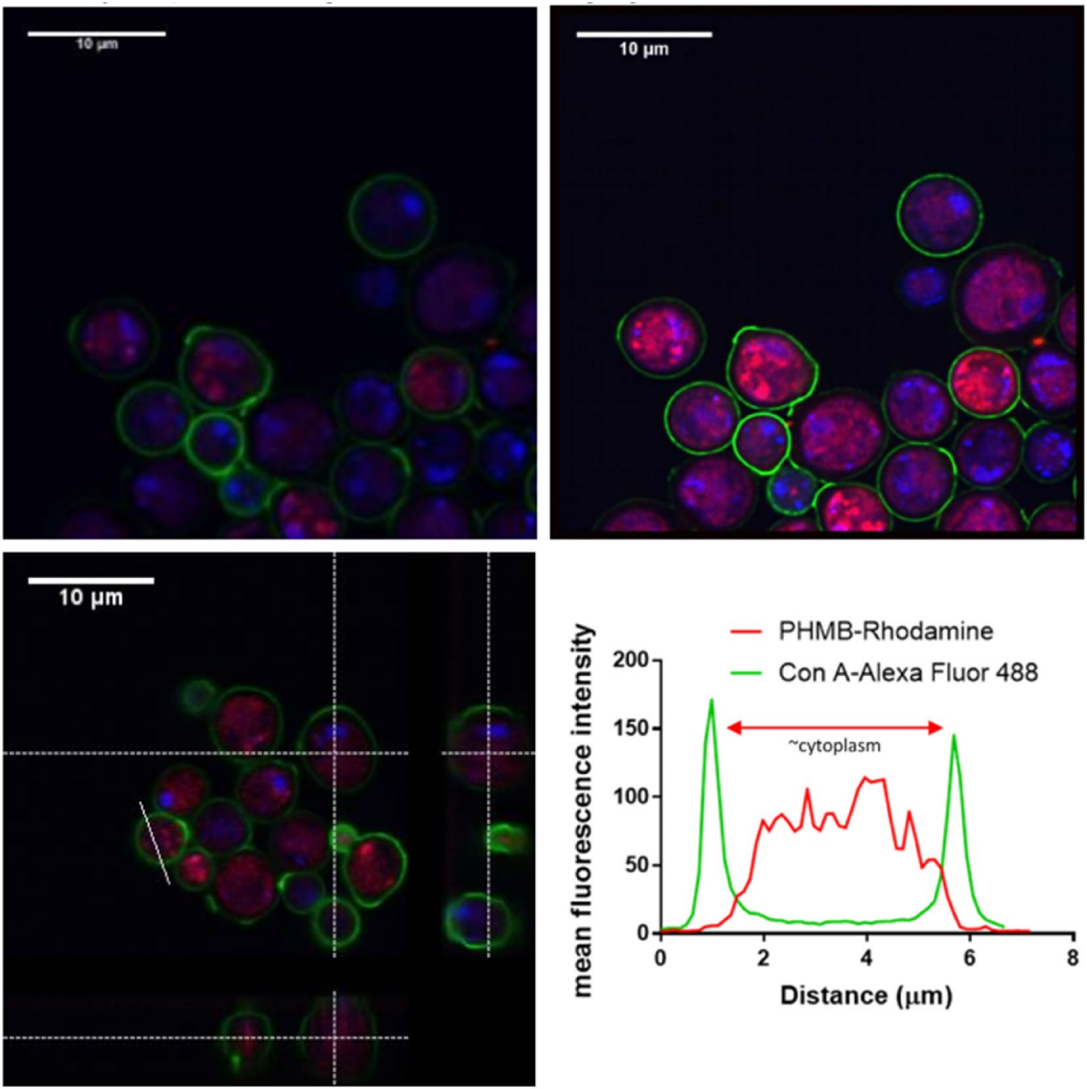
Confocal imaging of *S. cerevisiae* incubated with PHMB. *S. cerevisiae* were treated with PHMB-rhodamine (4 µg/ml) for 4 h at room temp. Cells were counterstained with DAPI and Con A-Alexa Fluor 488 and imaged by confocal microscopy. Top panels: Confocal images before (left) and after (right) image processing by SRRF. Bottom-left panel: Cross-sectional view of confocal Z-stacks of *S. cerevisiae* (59 slices). Images show PHMB-rhodamine accumulation within the cytosol and co-localisation with the nucleus (DAPI). The graph confirms high intracellular accumulation of PHMB-rhodamine.

**Figure 7:**
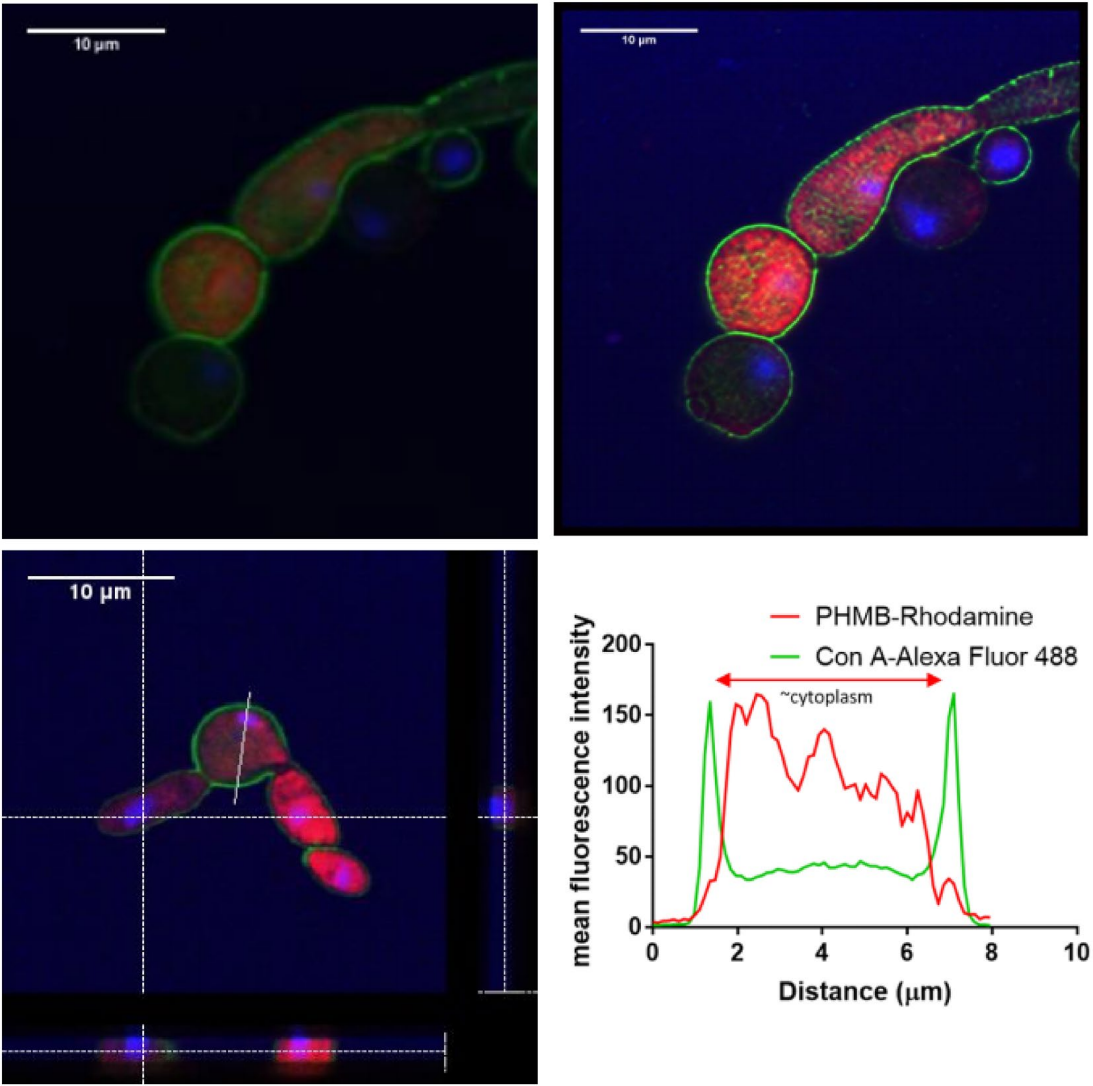
Confocal imaging of *C. albicans* incubated with PHMB. *C. albicans* were treated with PHMB-rhodamine (4 µg/ml) for 4 h at room temp. Cells were counterstained with DAPI and Con A-Alexa Fluor 488 and imaged by confocal microscopy. Top panels: Confocal images before (left) and after (right) image processing by SRRF. Bottom-left panel: Cross-sectional view of confocal Z-stacks of *C. albicans* (35 slices). Images show PHMB-rhodamine accumulation within the cytosol and co-localisation with the nucleus (DAPI). The graph confirms high intracellular accumulation of PHMB-rhodamine.

Confocal image analysis was performed on fixed yeast cells to confirm intracellular localisation. Cross sections of the yeast cells taken by Z-stack imaging confirms intracellular accumulation of PHMB within both *S. cerevisiae* and *C. albicans* (Figs. 6, 7). Plot analyses also show the presence of PHMB on the cell membrane at lower concentrations compared to the cytoplasm for both species. Nuclear localisation of PHMB in *C. albicans and S. cerevisiae* was apparent in the majority of cells. Furthermore, DAPI staining was weaker compared to other cellular stains which may be due to nuclear disruption as not all nuclei were intact. Therefore, PHMB appears to enter cells and localise within the cytoplasm and nucleus.

## Discussion

Polyhexamethylene biguanide is a cationic polymer with broad-spectrum antimicrobial activities. Previous studies to elucidate the antimicrobial mechanisms of PHMB have focused on its antibacterial mechanism^13,14,17^. Initially, the antibacterial mechanism was thought to be mediated through cell membrane disruption^13,14^. However, the current understanding involves PHMB entry into the bacteria cells where the polymer subsequently binds to and condenses bacterial chromosomes resulting in cell division arrest and death^17^. Thus, in this study, we sought to elucidate the polymer’s antifungal mechanism.

The MICs of PHMB, PHMB-rhodamine, positive control Triton x-114 and negative control terbinafine were first sought to determine suitable concentrations of drugs to be used for all membrane permeabilisation and imaging assays. The SYTOX green assay was used to determine the importance of cell membrane permeabilisation as an initial step in four fungal species (*S. cerevisiae*, *C. albicans*, *F. oxysporum*, *P. glabrum*) (Fig. 1). SYTOX green is a cationic dye that is excluded from healthy cells but is able to gain cell entry upon membrane permeabilisation by other agents, where it binds to DNA to generate a fluorescent signal. The observed cell membrane permeabilisation appeared to be dependent on both exposure time and PHMB concentration. The yeast species (*S. cerevisiae* and *C. albicans*) in particular, showed substantial increases in PHMB cell permeabilisation at 15 min and subsequent fluorescence following PHMB treatment at MIC_50_ in comparison to the filamentous fungi (*F. oxysporum*, *P. glabrum*) (Fig. 1)_._ Fluorescence intensity also appeared to peak at PHMB concentrations near their respective MIC_50_ values for all fungal species, followed by a loss in fluorescence signal (Figs. 2, 4). This sudden loss of fluorescence near and above MIC_90_ has also previously been observed in bacteria^17^. It suggests that membrane permeabilisation is not the polymer’s only mechanism of action as an increase in membrane permeabilisation should yield an increase in SYTOX Green:DNA fluorescence. The loss of fluorescence is likely due to competitive binding because like SYTOX green, PHMB has the ability to bind to DNA^16^. Thus, at higher concentrations, PHMB enters the fungal cell nucleus and binds to DNA, preventing SYTOX green binding, quenching the measured fluorescence signal.

As mentioned previously, the filamentous fungal species *F. oxysporum* and *P. glabrum* did not yield a significant increase in fluorescent signal following PHMB treatment at their MIC_50_ (Fig. 1). This observation was also noted for the detergent Triton X-114 positive control treated cells which elicits cidal effects via cell membrane disruption^26^. Thus, in accordance with the PHMB MIC of Table 1, susceptibility of fungi as well as the initial permeabilisation mechanism appears to be influenced by the accessibility of PHMB to the fungal cell surface. Yeast and filamentous fungi share overlapping but also distinct differences between their cell surface structures.

Generally, fungal cell walls are composed of two layers: an evolutionary conserved internal layer and a heterogeneous outer layer. The internal insoluble layer is comprised of carbohydrates including chitin, *β*-(1, 3)-glucan and *β*-(1,4)-glucan, required for the rigidity of fungal cell walls^27^. Whilst the outer layer is comprised mostly of glycosylated proteins, including mannose proteins, that are covalently linked to the *β*-(1,3)-glucan chitin matrix and also phosphorylated^28^. Phosphorylation of the chitin matrix confers an anionic charge to the fungal surface which would enable electrostatic interactions with cationic PHMB for cell wall destabilisation and subsequent cell membrane permeabilisation. This is shown in Figs. 2, 3 where PHMB permeabilises yeast fungal cell membranes over time.

In contrast, some filamentous fungi also contain *α*-(1,3)-glucan in the outer layer of the cell wall which is absent from the cell walls of *S. cerevisiae*, *C. albicans* and various other yeasts^27,28^. The presence of *α*-(1,3)-glucan in filamentous fungal cell walls has been shown to induce aggregation of germinating conidia in the *Aspergillus spp.* and *Penicillium spp.*^29^. Furthermore, the presence of an extracellular matrix might confer “biofilm like” protection which may delay PHMB’s ability to access the cell membrane to exert its antifungal effects^30^.

As PHMB uptake into fungi was influenced by exposure time and concentration, the uptake rate of the polymer as well as the exposure time required for complete permeabilsation at 30 °C was determined for *S. cerevisiae*, *C. albicans* and *F. oxysporum* (Fig. 5, Table 2). The uptake rate at 30 °C was calculated to be 0.03775 μg/ml min^−1^, 0.03177 μg/ml min^−1^ and 0.04607 μg/ml min^−1^ for *F. oxysporum*, *S. cerevisiae* and *C. albicans* respectively (Table 2). Cell membrane permeabilisatiion begins rapidly at time 0 for all fungal species at PHMB concentrations above MIC_90_. The time required for maximum permeabilsation at PHMB MIC_50_ was significantly higher for *F. oxysporum* compared to the yeast species. As mentioned previously, this is likely due to polymer binding to the extraceullar matrix of filamentous fungal species, effectively reducing the local concentration of PHMB at the fungal membrane.

To confirm intracellular accumulation of PHMB, confocal image analysis was performed on fixed *S. cerevisiae* and *C. albicans*. Cross sections of the yeast cells taken by Z-stack imaging confirmed intracellular accumulation of PHMB within both *S. cerevisiae* and *C. albicans* (Figs. 6, 7). The nucleus also appeared fragmented with an accompanied reduction of DAPI staining. PHMB’s binding affinity to DNA for chromosome condensation has previously been observed in prokaryotes where DNA is more accessible within the cytoplasm^7,17^. In contrast, in mammalian eukaryotic cells; PHMB is kept from the nuclear envelope, stored within endosomes and discarded^7^. Thus, despite the shared eukaryotic classification of mammalian and fungal cells, the polymer makes a mechanistic distinction between microbial and non-microbial eukaryotic cells to facilitate cell entry*.* This distinction may be due to differences in fungal and mammalian membrane lipid composition. The sequestration of PHMB into mammalian cell endosomes and its subsequent removal explains why there is a large therapeutic window between the in vitro antifungal effects and general cytotoxic effects.

It is generally accepted that early endosomes share the same lipid composition as their cell membranes. Mammalian endosomal membranes are composed of phosphatidylcholine (> 50%, no net charge), phosphatidylethanolamine (no net charge), phosphatidylserine (−ve charge), phosphatidylinositol (−ve charge) and phosphatidic acid (−ve charge)^31^. Although fungal endosomal membranes are also composed of these phospholipids, they occur in different proportions. For example, *S. cerevisiae* cell membranes are higher in phosphatidylserine (~ 30%) and phosphatidylinositol (~ 27%) and therefore possess a stronger net negative membrane charge^32^. This suggests that the antimicrobial distinction of PHMB may be driven by the strength of the electrostatic interactions between the cationic polymer and anionic phospholipids.

Furthermore, plot analyses (Figs. 6, 7) also showed the presence of PHMB on the cell membrane at lower concentrations compared to the cytoplasm for both species. The low membrane accumulation of PHMB further compounds the assertion that cell membrane permeabilisation is not the sole antifungal mechanism. Generally, membrane accumulation is observed with membrane permeabilising agents such as amphotericin B and other membrane permeabilising drugs which has not been observed here^33^.

**Figure 8:**
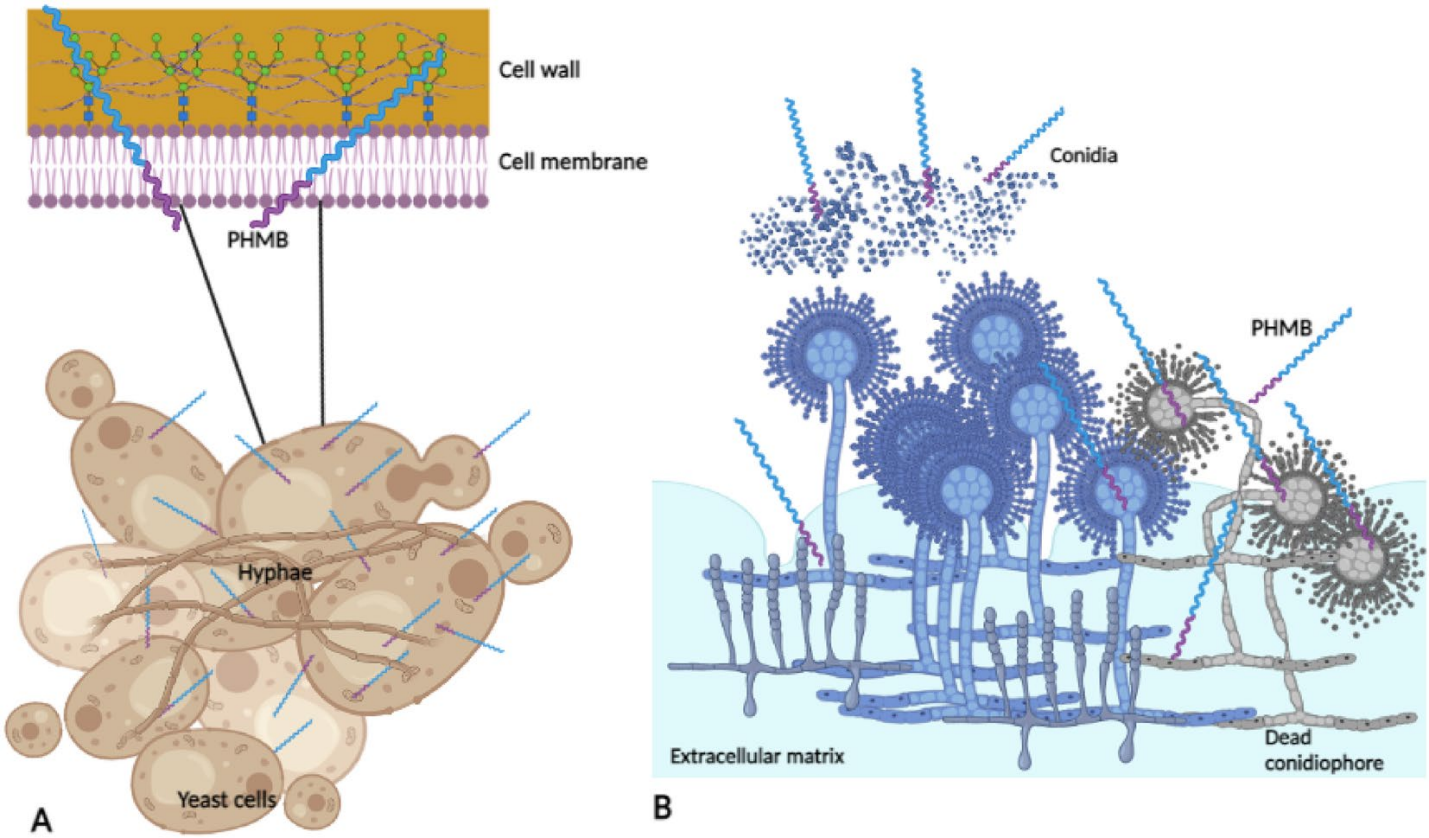
Schematic of varying susceptibilities of yeasts and filamentous fungi to PHMB. (**A**) Yeast cells are more susceptible to PHMB attack as the cell wall is anionic, enabling for the polymer’s adhesion. In addition, during budding, the *β*-(1,3)-glucan and chitin matrix are exposed which could facilitate PHMB cell entry. (**B**) Filamentous fungi appear to be less susceptible to PHMB attack due to the presence of the extracellular matrix (ECM). The ECM confers biofilm like protection to “mop up” PHMB by binding the polymer effectively; reducing its local concentration at the fungal cell membrane. PHMB penetration and hyphae/conidiophore accessibility. However, α-1,3 in the cell walls is exposed during conidia germination which increases the negative charge of conidia, thus increasing the cidal activity of cationic PHMB.

Consequently, PHMB appears to freely enter fungal cells via membrane permeabilisation without being trapped in endosomes (Fig. 8). Upon cell entry, it accumulates within the cytoplasm where it subsequently disrupts the nucleus and binds to DNA. The observed nuclear disruption suggests that there are other potential intracellular targets of PHMB as the nuclear membrane and endoplasmic reticulum membrane are continuous. In addition, like bacteria, yeast mitochondria is also prokaryotic in origin. Therefore, the potential interaction of PHMB with other organelles requires further investigations.

## Conclusion

PHMB gains access to the fungal cell membrane where it interacts with the cell membrane through electrostatic interactions and begins the process of permeabilisation. Following cell membrane disruptions, it accumulates within the cytosol where it disrupts the nuclear membrane and binds to DNA for further fragmentation. Although four fungal species were analysed, they belong to two large fungal families namely *Saccharomycetaceae* and *Nectriaceae*. Thus, their observed interactions with PHMB are reflective of a much wider fungal genus. This study provides a better understanding of PHMB’s non-specific mechanism of action as an alternative antifungal agent with low risk of resistance. However, the extent of fungal cell wall vulnerability and interaction with other organelles remains to be assessed.

## Data Availability

The datasets generated during and/or analysed during the current study are available from the corresponding author on reasonable request.
